# Altered interhemispheric synchrony in Parkinson’s disease patients with levodopa-induced dyskinesias

**DOI:** 10.1038/s41531-020-0116-2

**Published:** 2020-07-08

**Authors:** Caiting Gan, Min Wang, Qianqian Si, Yongsheng Yuan, Yan Zhi, Lina Wang, Kewei Ma, Kezhong Zhang

**Affiliations:** 1 Department of Neurology, The First Affiliated Hospital of Nanjing Medical University, No. 300 Guangzhou Road, Nanjing, 210029 China; 2 Department of Radiology, The First Affiliated Hospital of Nanjing Medical University, No. 300 Guangzhou Road, Nanjing, 210029 China

**Keywords:** Parkinson's disease, Parkinson's disease, Parkinson's disease

## Abstract

Levodopa-induced dyskinesias are common motor complication of Parkinson’s disease after 4–6 years of treatment. The hallmarks of dyskinesias include unilateral onset and the tendency to appear on the more affected body sides. There is a growing literature documenting the lateralization abnormalities are associated with the emergence of dyskinesias. Our investigation aimed to explore interhemispheric functional and its corresponding morphological asymmetry. A total of 22 dyskinetic patients, 23 nondyskinetic patients, and 26 controls were enrolled. Resting-state functional magnetic resonance imaging scans were performed twice before and after dopaminergic medication. Voxel-mirrored Homotopic Connectivity (VMHC) and Freesurfer were employed to assess the synchronicity of functional connectivity and structural alternations between hemispheres. During OFF state, dyskinetic patients showed desynchronization of inferior frontal cortex (IFC) when compared to nondyskinetic patients. And during ON state, dyskinetic patients showed desynchronization of IFC and pre-supplementary motor area (pre-SMA) when compared to nondyskinetic patients. However, there was no corresponding significant asymmetries in cortical thickness. Moreover, the degree of desynchronization of IFC and pre-SMA in dyskinetic pateients during ON state were negatively correlated with the Abnormal Involuntary Movement Scale (AIMS) scores. Notably, among patients who showed asymmetrical dyskinesias, there was a significant negative correlation between VMHC values of IFC and dyskinesias symptom asymmetry. Our findings suggested that uncoordinated inhibitory control over motor circuits may underlie the neural mechanisms of dyskinesias in Parkinson’s disease and be related to its severity and lateralization.

## Introduction

Levodopa-induced dyskinesias (LIDs), which are recognized as involuntary, principally chorea movements^1^, occur in 40% of patients with Parkinson’s disease (PD) after 4–6 years of levodopa treatment^2^. Dyskinesia is most common at the peak-level of levodopa action which often coincides with high plasma levodopa levels^3^. The characteristics of LIDs include unilateral onset and the tendency to appear on the more affected body sides, showing the asymmetry in clinical presentation on the two sides of body^4^. For example, peak-dose levodopa-induced dyskinesia tends to involve the upper trunk, neck, and arms, particularly on the more affected side^4^. Recently, many structural and functional neuroimaging studies have attempted to discover the underlying mechanism of dyskinesias. Among them, some researchers noticed that there was pronounced increased cortical thickness and underactivity of the right side of inferior frontal cortex (IFC), while some literature showed bilateral functional or structural changes, with respect to patients without LIDs^5–7^. Also, another cohort manifested that higher striatal asymmetric index was more susceptive to develop dyskinesia^8^. Although these previous findings have preliminary results, the role of the interhemispheric functional asynchronization and its corresponding morphological asymmetry in the pathophysiology of LIDs still remains unclear.

Thus, this study aimed to discover abnormalities in the functional coordination between hemispheres in LIDs. To do so, we adopted a method called Voxel-mirrored Homotopic Connectivity (VMHC), measuring the functional connectivity between geometrically corresponding interhemispheric regions^9^. Homotopic functional connectivity is sensitive to detect interhemispheric coordination alterations and may mirror the consequence of interhemispheric communication to integrated brain function underlying coherent behavior and cognition. Although it is the first use in the study on LIDs, VMHC has been widely and successfully applied to explore the interhemispheric functional coordination in a variety of neurological and psychiatric diseases (e.g., schizophrenia, Parkinson’s disease, Alzheimer’s disease, amyotrophic lateral sclerosis, depression, traumatic axonal injury)^10–15^. Furthermore, we investigated the corresponding cortical thickness asymmetry of these regions to probe whether the changes in interhemispheric functional coordination resulted from alterations in anatomic coordination. And we hope the exploration of the pathologic mechanism of dyskinesia would provide possible therapeutic target for neuromodulation (e.g. repetitive transcranial magnetic stimulation) for the clinical treatment of dyskinesia.

## Results

### Demographic and clinical characteristics

Demographic and clinical characteristics of the participants are summarized in Table 1 and Supplementary Table 1. No significant differences were found among the three groups in terms of age, gender, or education levels. However, dyskinetic group had a longer duration of disease (*p* = 0.017) compared with nondyskinetic group. Hence, it would be included as one of the nuisance variables when comparing dyskinetic and nondyskinetic PD patients. In addition, there was no difference in age at onset, Unified Parkinson’s Disease Rating Scale section III (UPDRS-III) ON and OFF period, Hoehn and Yahr (H&Y) staging ON and OFF state, and levodopa equivalent daily dose (LEDD) between groups.

**Table 1:** Demographic and clinical characteristics of all subjects.

Variables	Dyskinetic	Nondyskinetic	Controls	*P* values
n	22	23	26	NA
Gender (F/M)	10/12	8/15	8/18	0.561^a^
Age (y)	63.31 ± 7.39	61.91 ± 8.22	63.50 ± 5.19	0.695^b^
Education (y)	8.59 ± 3.25	7.30 ± 2.69	8.50 ± 2.25	0.209^b^
Age at onset (y)	53.50 ± 9.62	56.13 ± 7.22	NA	0.304^c^
Disease duration (y)	9.14 ± 4.97	6.13 ± 2.91	NA	0.017^d,*^
Hoehn and Yahr stage ON phase	2.09 ± 0.59	1.78 ± 0.69	NA	0.082^d^
Hoehn and Yahr stage OFF phase	2.64 ± 0.66	2.22 ± 0.77	NA	0.057^d^
UPDRS-III ON phase	21.91 ± 8.18	19.22 ± 14.68	NA	0.455^c^
UPDRS-III OFF phase	39.23 ± 16.52	34.04 ± 17.43	NA	0.312^c^
LEDD	795.02 ± 310.29	750.38 ± 426.27	NA	0.237^d^
MMSE	27.77 ± 1.51	28.00 ± 1.86	NA	0.338^d^
AIMS	10.54 ± 5.21	NA	NA	NA

Values are presents as the mean ± standard deviation.

*NA* not applicable, *F* Female, *M* Male, *y* year, *UPDRS* unified Parkinson’s disease rating scale, *LEDD* Levodopa equivalent daily dose, *MMSE* Mini-Mental State Examination, *AIMS* abnormal involuntary movement scale.

^a^Chi square test.

^b^One-way analysis of variance.

^c^Two-sample t-test.

^d^Mann–Whitney test.

### VMHC findings and analysis

**Fig. 1:**
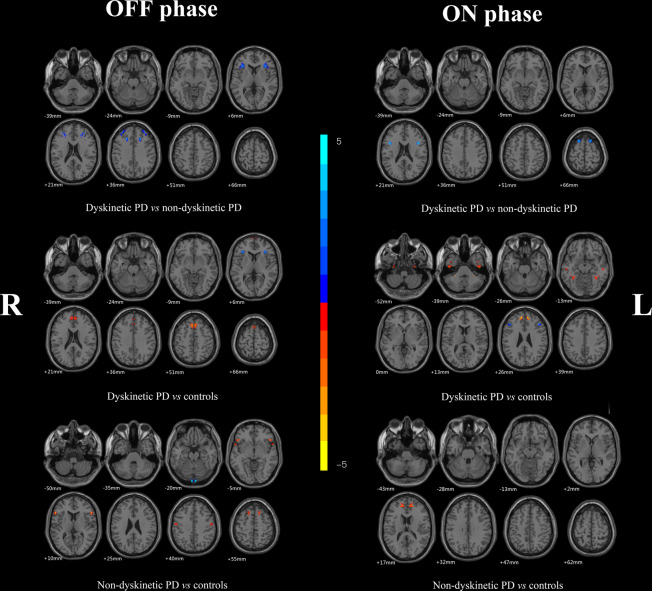
Statistical maps showing VMHC differences in different brain regions between three groups during ON and OFF phase, respectively. The threshold for display was set to *p* < 0.01.

Significant differences of the VMHC values among the three groups during the OFF or ON state were observed by an analysis of covariance (ANCOVA) analysis, followed by the two-sample post hoc t-test (Fig. 1; Table 2). During OFF phase, dyskinetic group showed reduced VMHC values in the triangular part of IFC, compared to nondyskinetic group, adjusting for age, gender, education, mean FD, and disease duration. Compared with HCs, PD patients with dyskinesias had decreased VMHC in the triangular part and increased VMHC in pre-SMA and supplementary motor area (SMA). Meanwhile, the PD patients without dyskinesias had higher VMHC values in the superior temporal gyrus (STG), postcentral gyrus (PoCG), and pre-SMA relative to the HC group.

**Table 2:** VMHC differences among dyskinetic PD patients, nondyskinetic PD patients and health controls during ON and OFF phase.

Brain regions	Number of voxels	MNI coordinates			*T* value
		*X*	*Y*	*Z*	
*OFF phase*					
Dyskinetic vs Nondyskinetic					
IFC (pars triangularis)	177	±33	30	9	−4.6641
Dyskinetic vs Controls					
SMA	49	±6	24	48	4.3726
IFC (pars triangularis)	20	±33	30	9	−4.0024
Pre-SMA	36	±6	48	24	3.3655
Nondyskinetic vs Controls					
STG	65	±57	12	−3	3.7929
PoCG	73	±54	−18	45	3.7058
Pre-SMA	27	±15	30	36	3.5218
*ON phase*					
Dyskinetic vs Nondyskinetic					
IFC (pars triangularis)	13	±42	12	21	−3.8266
Pre-SMA	40	±19	17	65	−3.8087
Dyskinetic vs Controls					
FFG	20	±36	−45	−15	3.3327
MTG	10	±54	−21	−15	3.4031
IFC (pars triangularis)	14	±45	30	27	−3.2272
Pre-SMA	10	±9	48	27	3.5466
Nondyskinetic *vs* Controls					
Pre-SMA	27	±12	45	15	3.2503

A corrected threshold of *p* < 0.01 corrected by Monte Carlo.

*MNI* Montreal Neurological Institute, *IFC* inferior frontal cortex, *SMA* supplementary motor area, *Pre-SMA* pre-supplementary motor area, *STG* superior temporal gyrus, *PoCG* postcentral gyrus, *FFG* fusiform gyrus, *MTG* middle temporal gyrus.

After taking their current dopaminergic medication, all PD patients expressed increased VMHC in pre-SMA when compared with HCs. The dyskinetic group showed significantly reduced VMHC values in the triangular part and pre-SMA compared with nondyskinetic group, adjusting for age, gender, education, mean FD and disease duration. In addition, the increased VMHC of middle temporal gyrus (MTG) and fusiform gyrus (FFG) and decreased VMHC of IFC was also observed in the LID patients relative to the HCs.

**Fig. 2:**
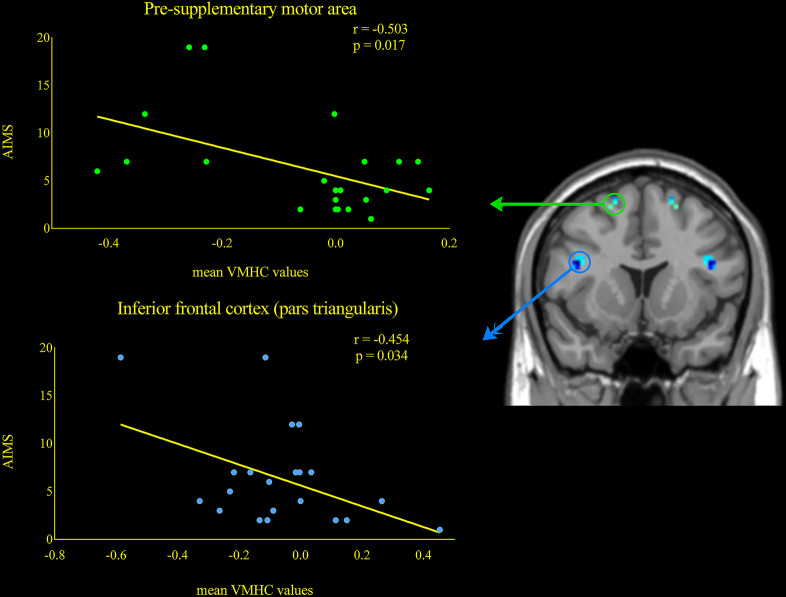
Correlations between VMHC values and AIMS scores during ON phase within the LIDs patients. Scatterplots demonstrated that there was a significant negative correlation between the mean VMHC values in the pre-supplementary motor area and AIMS scores in LIDs patients. Besides, a negative correlation was also found between the mean VHMC values in the inferior frontal cortex (pars triangularis) and AIMS scores. Abbreviations: VMHC: voxel-mirrored homotopic connectivity; AIMS: Abnormal Involuntary Movement Scale.

**Fig. 3:**
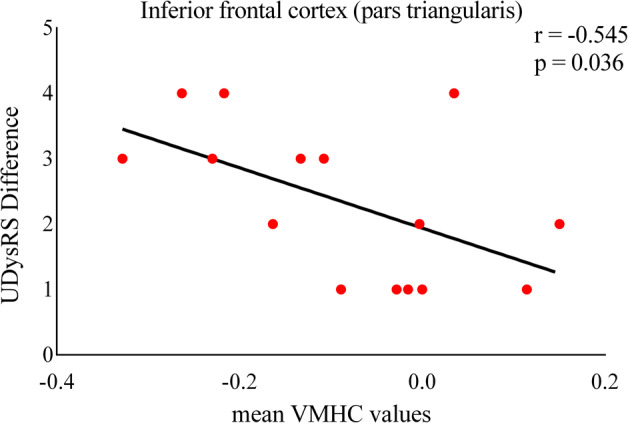
Relation between LIDs symptoms asymmetry and VMHC values of IFC (pars triangularis) during ON phase. Asymmetry of dyskinesias symptoms was determined by calculating the absolute value of the difference between the left and right scores evaluated by UDysRS-III subscores. There was a significant relationship between dyskinesias symptoms asymmetry and desynchronization of inferior frontal cortex (pars triangularis) in the two hemispheres. Abbreviations: VMHC: voxel-mirrored homotopic connectivity; UDysRS: Unified Dyskinesia Rating Scale.

Furthermore, AIMS scores were inversely correlated with VMHC of IFC (pars triangularis) (*r* = −0.503, *p* = 0.034) and pre-SMA (*r* = −0.454, *p* = 0.017) (Fig. 2) in dyskinetic PD patients during ON state. These indicated that the more severe symptoms of dyskinesias, the worse coordination of IFC and pre-SMA. Finally, we found a significant correlation between the degree of desynchronization of IFC (pars triangularis) and dyskinesias symptom asymmetry (*r* = −0.545, *p* = 0.036) (Fig. 3), suggesting that with the deepening of asymmetry symptom, the function coordination of IFC will be getting poorer. These indicated that the more severe symptoms of dyskinesias, the worse function coordination of IFC and pre-SMA. No significant association was found between VMHC values and illness duration, UPDRS-III score (ON and OFF), H&Y scale score (ON and OFF), Mini-mental State Examination (MMSE) and LEDD.

### Cortical thickness asymmetry analysis

No significant change in the asymmetry index (AI) of cortical thickness was found in the brain regions that showed significant differences in VMHC analysis at a threshold of Bonferroni-corrected *p* < 0.05 (Supplementary Table 2).

## Discussion

In this study, we investigated the interhemispheric functional and structural coordination among dyskinetic, nondyskinetic PD patients and healthy controls by combining VMHC and Freesurfer approaches. Our primary finding was that dyskinetic patients showed significantly reduced VMHC values in IFC and pre-SMA during ON phase and exhibited lower VMHC values in IFC during OFF phase, when compared to nondyskinetic patients. However, there was no cortical thickness asymmetry in corresponding brain regions. Thus, we speculated that interhemispheric brain function desynchronization was not associated with structural changes, or that the alternation of functional synchronization in LIDs had not led to the structural lateralization. Furthermore, significant negative correlations were observed between the mean VMHC values of pre-SMA and IFC (pars triangularis) during ON phase and AIMS scores in the LIDs group. And VMHC values within IFC during ON phase were negatively correlated with symptom asymmetry in patients who displayed asymmetrical dyskinesias.

Classically, LIDs are attributable to the degree of nigrostriatal neurodegeneration and striatal alternation related to chronic levodopa therapy, which may induce plastic synaptic abnormalities in striatal medium spiny neurons and further have an impact on alternation of neuronal activity in striato-pallidal circuits^16^. With more and more studies focused on LIDs, the pathophysiological mechanisms have been modified from striato-motor circuitry to a broader cortico-cortical network, involving frontal cortex (including pre-SMA and IFC)^6,17–19^. As is generally known that IFC is critical for response inhibition through sending a stop command to primary motor cortex (PMC) (M1)^20^. A previous study provided direct evidence that IFC/M1 interactions were involved in cortico-cortical and subcortical pathways during action inhibition^21^. The detected underactivation of IFC in LIDs group represented the decrease of inhibitory control, and further would lead to the emergence of involuntary movement^22^, which was usually evaluated by AIMS clinical scale (higher values represent greater motor complications). Moreover, we further discovered that the more severe degree of desynchronization of IFC, the more obvious lateralization of dyskinesia symptoms, which suggesting the imbalance in the pathological progression of bilateral IFC might play a key role in the pathophysiological mechanisms of LIDs. And our abnormal finding suggested chronic levodopa treatment would lead abnormal functional connectivity in certain brain regions that can be observed after several hours off levodopa medication. This phenomenon may be explained by long-term remodeling or abnormal recombination of neuronal contacts or pathways in IFC^5^.

Another important finding in our study was the altered VMHC of pre-SMA during ON phase with the presence of clinical dyskinesias. As we know, pre-SMA has been identified as indispensable in stopping for its role in switching between tasks, and between rules linking stimuli to responses^23,24^. Studies have revealed that this two frontocortical areas work differently in stop-signal inhibition, with the IFC mediating attentional processing of the stop signal and the pre-SMA mediating response inhibition^25^. From Li et al.^26^, although both the pre-SMA and IFC are recruited in successful inhibition, only the pre-SMA is important for more efficient stopping. Granger causality analyses showed that pre-SMA and PMC had strong interconnectivity with the basal ganglia circuitry of motor control to determine the outcome of go and stop processes, whereas the IFC indirectly influenced the basal ganglia circuitry via connectivity with pre-SMA. This result represented that the inhibition of IFC was dependent on pre-SMA^25^. Thus, we may hypothesize that the decrease in IFC alone would not lead to the occurrence of dyskinesias, but only when IFC and pre-SMA decreased simultaneously. And we speculated that the imbalance in the pathological progression in the IFC, might have led to the desynchronization of pre-SMA in the two hemispheres. In addition, Lee et al. observed the right pre-SMA’s efficiency in updating motor planning and reinitiating a partially inhibited response in a conditional stop-signal task by applying transcranial magnetic stimulation^27^. In general, the absence of pre-SMA inhibition and the uncoordinated function of bilateral IFC might be more important mechanism to induce dyskinesias. Consecutive studies, however, were also reported opposite findings, namely an increase of pre-SMA activity in LIDs^18,28^. These inconsistencies might partly be due to the preprocessing and statistical analysis of fMRI data, and the extent to which fMRI research can contribute to our understanding of the abnormal neural mechanisms of dyskinesias in PD remains to be clarified.

Compared with HCs, PD patients showed increased VMHC values in pre-SMA both in ON and OFF state, which was in keeping with the previous studies^29–31^. Herz et al^19^. explained that PD patients tend to pay extra attention to their actions “by default”, even if this is not explicitly required. PD patients need the overactivity of pre-SMA to achieve near-normal performance. In order to attain this, the homotopic areas in both hemispheres had increase functional connectivity to enhance the normally lateralized activation network^32^.

Nevertheless, the current experiment has some other limitations in addition to the relatively insufficient sample size. First, the brain is not absolutely structurally symmetrical. Hence, we applied a symmetric temple to resolve this problem. Besides, AI values in brain regions that might be critical to the onset of dyskinesias were further analyzed. Second, VMHC has its methodological limitations that it cannot study functional connectivity within the cerebral hemisphere or determine which side of the brain is damaged. Third, it is difficult to determine the precise time of peak-dose effects emerging during our fMRI investigation. And PD patients may be biased in their perception of when the drug will take effect. Besides, we investigated LID patients before they came up to peak-of-dose dyskinesias, which was incompatible with fMRI study. Therefore, we have no idea whether this connection pattern would become more evident during the peak-dose phase.

To sum up, the decreased VMHC values within the bilateral IFC and pre-SMA may reflect uncoordinated inhibitory control over motor circuits that induced the emergence of involuntary movement, especially the IFC might be associated with dyskinesias lateralization. Our study may provide a new insight to the pathophysiological mechanisms of dyskinesias.

## Methods

### Subjects

Our initial sample consisted of 50 right-handed patients with diagnosis of idiopathic PD judging by the UK Parkinson’s Disease Society Brain Bank criteria recruited from the Neurology Unit of the First Affiliated Hospital of Nanjing Medical University. Inclusion criteria were: (1) unilateral onset of PD; (2) no family history of PD; (3) a minimum 6-month duration of levodopa therapy; (4) stable medication dose for 4 weeks at least; (5) presence or absence of peak-dose LIDs after following an acute levodopa test observed by the examining neurologist on the occasion of the last visit; (6) no contraindications of magnetic resonance imaging (MRI) scans; (7) no evidence of brain anatomical abnormalities; (8) no evidence of global cognitive impairment (MMSE score > 24); (9) no use of antidepressant, anxiolytic, or antipsychotic drugs that could affect cerebral functional change; (10) basic motor performance and no excessive movement artifacts (head motions more than 2.0 mm of translation or 2.0° of rotation) during fMRI experiment; and (11) ability to tolerate the withdrawal of dopaminergic medication before functional MRI session. According to the above criteria, one patient was not able to undergo the MRI scans because he was hard to bear the withdrawal of dopaminergic medication. After careful screening of MRI data, four patients were excluded from the study because of head movement artifacts (details later).

Ultimately, 22 PD patients with LIDs and 23 PD patients without LIDs were enrolled. All dyskinetic patients exhibited peak-dose choreic dyskinesias, rather than off-period LIDs or diphasic LIDs, predominately distributed in the upper body or lower extremities. Among LIDs group, dyskinesias first appeared on the ipsilateral side of the onset of PD motor symptoms, and 15 dyskinetic patients showed asymmetrical dyskinesias symptoms during the evaluation, whose scores on the left (Item 18 + 21) and right (Item 19 + 22) sides of Unified Dyskinesia Rating Scale part III (UDysRS-III) were not equal, as illustrated in Supplementary Table 1. Additionally, a group of 26 gender- and age-matched healthy controls (HCs) was also recruited.

This study was approved by the ethics committee of the First Affiliated Hospital of Nanjing Medical University, and all participants gave their informed written consent before beginning the experiment.

### Study design

All patients underwent identical experimental steps, scanned twice in the same morning. In line with a previous study^33^, we estimated the rough time of LIDs onset one week before the experiment on account of the patient’s past medication experience, and patients were required to keep a diary of when drugs worked and out of work for a week before scans. Meanwhile, in order to obtain the average time for each dyskinetic individual transforming from an “OFF” state to an “ON” state, patients who were after a night withdrawal of anti-parkinson drugs, were prescanned after and before taking their current dopaminergic medication regimes. This above information was applied to start fMRI image acquisition before patients developed LIDs during experiments. The first scan (practical “OFF” phase) of our experiment was acquired after 12 h withdrawal of all dopaminergic medication. Then patients took their usual morning levodopa dose and the second MRI scan (“ON” phase) was performed when the patients responded to levodopa as expected. Meanwhile, during “OFF” and “ON” phase, patients were evaluated by clinical assessments, including H&Y staging, UPDRS-III, AIMS and UDysRS-III. MMSE was also assessed to exclude cognitive impairment. In addition, we arranged for a professional neurologist to remain in the scanning room and observed the appearance of involuntary movements. MRI scans were ceased instantly as long as dopaminergic levels reached the critical point triggering dyskinesias. At this period, none of the dyskinetic PD patients came up to dyskinesias.

### Image acquisition

All participants were scanned by a 3.0 T Siemens MAGNETOM Verio whole-body MRI system (Siemens Medical Solutions, Germany) equipped with eight-channel, phase-array head coils. Three-dimensional T1-weighted anatomical images were acquired using the following volumetric 3D magnetization-prepared rapid gradient-echo (MP-RAGE) sequence with the following parameters: repetition time [TR] = 1900 ms, echo time [TE] = 2.95 ms, flip angle [FA] = 9°, slice thickness = 1 mm, slices = 160, field of view [FOV] = 230 × 230 mm^2^, matrix size = 256 × 256 and voxel size = 1 × 1 × 1 mm^3^. Resting-state functional images were collected using an echo-planar imaging (EPI) sequence with the following parameters: TR = 2000 ms, TE = 21 ms, FA = 90°, FOV = 256 × 256 mm^2^, in-plane matrix = 64 × 64, slices = 35, slice thickness = 3 mm, no slice gap, voxel size = 3 × 3 × 3 mm^3^, total volumes = 240. During the scanning process, all subjects were instructed to close their eyes, keep awake, remain motionless, and not to think about anything in particular.

### Preprocessing of fMRI data analysis and VMHC

Rs-fMRI data preprocessing was performed on Data Processing Assistant for Resting-State fMRI (DPARSF, http://www.restfmri.net/forum/dparsf) and REST (http://restfmri.net). The preprocessing procedures can be briefly divided into the following steps. The first 10 time points were discarded and the remaining 230 images were corrected for timing differences between slices and head motion (Friston 24 parameter). Four participants (3 dyskinetic PD patients and 1 nondyskinetic PD patient) with head motions more than 2.0 mm of translation or 2.0° of rotation were excluded. Subsequently, individual T1 structural images were co-registered to the mean EPI scans and segmented into gray matter and white matter using “New Segment”. DARTEL normalization was applied to compute the transformations from the native space to the Montreal Neurological Institute (MNI) space. The following steps comprised spatial normalization of the EPI images using the transformation parameters assessed in the previous preprocessing procedures, re-sampling with 3 × 3 × 3 mm^3^ resolution, and spatially smoothed with a 6 mm full width half maximum Gaussian kernel to decrease spatial noise. The resulting fMRI data were linearly trend removed and temporally filtered (0.01–0.08 Hz). Several sources of spurious variance were regressed out, including the white matter signal, the cerebral spinal fluid signal, and six head motion parameters obtained by head-motion correction.

Further, we computed the mean framewise displacement (FD) for each subject and used it as a covariate in the following inter-group comparisons of VMHC. Also, the group comparisons of motion parameters showed no significant differences (*p* > 0.05).

For VMHC computation, first, a mean normalized T1 image was created by averaging the spatially normalized T1 images. Afterwards, this T1 image was averaged with its left-right mirrored version to create a symmetric brain template and the individual T1 images were registered non-linearly to this group-specific symmetric template. The identical transformation was applied to the resting-fMRI images. The homotopic functional connectivity for each subject was computed as the Pearson correlation coefficient between any pair of symmetric interhemispheric voxels. Correlation values were then Fisher z-transformed to improve the normality. The resultant values constituted the VMHC and were used for the group analyses. More details of VMHC data processing were described in a previous literature^8^.

### Cortical thickness

To test whether these VMHC findings were associated with structural changes, we further performed cortical thickness analysis within the brain regions showing significant differences among the three groups. The procedures carried out by FreeSurfer software included: removal of non-brain data, intensity normalization, tessellation of the gray-white matter boundary, automated topology correction and accurate surface deformation to identify tissue borders^34,35^. Results for all subjects were visually inspected to ensure accuracy of registration, skull stripping, segmentation, and cortical surface reconstruction. For each subject, cortical thickness measurement was calculated as the distance between the white matter and gray matter surfaces at each vertex of the reconstructed cortical mantle. The estimated total intracranial volume (ICV) was calculated and used as a covariate in the following group comparison.

As a measure of asymmetry for each bilaterally cortical area, we calculated the AI by the following formula^36^. The R and L stands for the values of cortical thickness of the corresponding cortical region from the right and left hemisphere, respectively.

$${\rm{AI}} = \frac{{|L - R|}}{{L + R}} \times {\mathrm{100}}.$$

We regarded that as a decrease in cortical asymmetry, when the values of AIs of a patient with LIDs or without LIDs were less than those of HCs.

### Statistical analysis

Differences among the three groups in terms of demographic and clinical variables were examined using Chi square test, one-way analysis of variance (ANOVA), two-sample t-test and Mann-Whitney test with SPSS 20.0 statistical analysis software (SPSS Inc. Chicago, IL, USA), as appropriate. Significance threshold was set to *p* = 0.05.

Group comparisons of VMHC were processed with REST software. ANCOVA was performed to identify brain areas with significant differences in VMHC among the three groups during OFF phase and ON phase, by adding age, gender, education and mean FD as covariates (OFF phase: voxel-level *p* < 0.01, cluster size > 34 voxels, corresponding to a corrected *p* < 0.01 as determined by AlphaSim correction; ON phase: voxel-level *p* < 0.01, cluster size > 37 voxels, corresponding to a corrected *p* < 0.01 as determined by AlphaSim correction). The post hoc two-sample t tests were conducted within a mask showing significant differences obtained from the ANCOVA analysis, with correction (OFF phase: voxel-level *p* < 0.01, cluster size > 20 voxels, determined by a Monte Carlo simulation resulted in a cluster-level significance threshold of *p* < 0.01; ON phase: voxel-level *p* < 0.01, cluster size > 9 voxels, determined by a Monte Carlo simulation resulted in a cluster-level significance threshold of *p* < 0.01). Brain regions showing significant differences between dyskinetic and nondyskinetic patients during ON state were selected as ROIs. Then, Pearson correlation coefficients were computed between the extracted mean VMHC values within the ROIs and the AIMS scores of patients with peak-dose dyskinesias. Finally, we calculated the correlation between the degree of desynchronization of ROIs among asymmetric dyskinetic patients and their symptom asymmetry. Symptom asymmetry was determined by calculating the absolute value of the difference between the left and right scores evaluated by the UDysRS-III subscores. Significance level was set at *p* < 0.05 (two-tailed).

The AIs of the brain regions showing significant differences among the three groups in VMHC analysis were compared by ANCOVA, with age, gender, education and ICV as covariates^36^. In the post hoc analyses, the significance level was adjusted by Bonferroni corrections.

## Supplementary information

supplementary material

Related Manuscript File

## Data Availability

The data that support the findings of this study are available from the corresponding author upon reasonable request.

## Code availability

As previously mentioned in the MRI subsection, the cortical thickness was analyzed using FreeSurfer (version 6.0). VMHC analysis was performed on Data Processing Assistant for Resting-State fMRI (DPARSF, http://www.restfmri.net/forum/dparsf) and REST (http://restfmri.net).
