# Supplementary material for: Altered interhemispheric synchrony in Parkinson’s disease patients with levodopa-induced dyskinesias
Source: NPJ Parkinsons Dis. 2020 Jul 8;6:14. doi: 10.1038/s41531-020-0116-2 (PMC7343784; doi:10.1038/s41531-020-0116-2)
Supplement: Supplementary file 1 — supplementary material [file 41531_2020_116_MOESM1_ESM.pdf]

Supplementary Table 1. Clinical details in dyskinetic PD patients.

Case	Disease duration (years)	Age/ sex	Side onset of PD	UDysRS Part III								
				Item 16 (face)	Item 17 (neck)	Item 18 (right shoulder/arm)	Item 19 (left shoulder/arm)	Item 20 (trunk)	Item 21 (right hip/leg)	Item 22 (left hip/leg)	Item 18+21	Item 19+22
1	3	51/M	R	0	0	0	0	2	2	2	2	2
2	6	63/F	L	0	0	0	2	0	0	2	0	4
3	13	56/M	R	1	0	3	0	2	0	2	3	2
4	12	75/M	L	0	0	0	2	3	0	2	0	4
5	5	55/M	L	3	3	3	3	3	0	0	3	3
6	7	73/F	R	1	1	2	2	2	2	2	4	4
7	7	67/M	L	0	0	1	1	2	1	1	2	2
8	7	54/F	L	0	0	1	2	0	0	0	1	2
9	13	67/M	L	0	0	0	2	1	0	1	0	3
10	10	61/M	R	2	0	2	2	1	1	1	3	3
11	10	64/F	L	0	1	1	2	0	1	2	2	4
12	25	64/F	L	2	1	2	3	2	3	3	5	6
13	9	72/M	L	0	0	1	3	1	1	3	2	6
14	13	62/F	R	0	0	2	0	0	0	0	2	0
15	15	65/F	L	0	0	2	1	0	1	1	3	2
16	2	65/M	R	0	0	2	0	0	1	0	3	0
17	9	65/F	L	0	0	0	3	1	0	0	0	3
18	10	51/M	L	0	2	0	3	0	0	0	0	3
19	9	75/M	R	0	0	3	2	2	2	1	5	3
20	4	61/F	R	0	0	1	1	0	1	1	2	2
21	7	72/F	R	0	0	2	1	2	0	0	2	1
22	5	55/M	L	2	2	3	3	3	0	0	3	3

Abbreviations: UDysRS-III: Unified Dyskinesia Rating Scale part III; F: Female; M: Male; R: Right; L: Left.

Supplemetary Table 2. Asymmetry index mean values of the brain region that showing significant differences of VMHC among three groups.

Brain regions	Asymmetry Index			
	Dyskinetic	Nondyskinetic	Controls	P values
IFC (pars triangularis)	2.53 ± 0.39	2.06 ± 0.38	2.47 ± 0.36	0.640
Pre-SMA	1.15 ± 0.17	1.03 ± 0.17	1.18 ± 0.16	0.804
FFG	1.65 ± 0.25	1.61 ± 0.25	1.55 ± 0.23	0.957
MTG	1.56 ± 0.26	1.82 ± 0.25	1.54 ± 0.24	0.672
STG	1.74 ± 0.24	1.22 ± 0.23	1.47 ± 0.22	0.291
PoCG	1.23 ± 0.32	1.53 ± 0.31	2.07 ± 0.30	0.155
SMA	2.05 ± 0.29	2.00 ± 0.28	1.73 ± 0.26	0.682

Values are represented as the mean ± standard deviation. The significance of ANCOVA was set at $p < 0.05$. Abbreviation: IFC: Inferior Frontal Cortex; Pre-SMA: Pre-supplementary Motor Area; FFG: Fusiform Gyrus; MTG: Middle Temporal Gyrus; STG: Superior Temporal Gyrus; PoCG: Postcentral gyrus; SMA: Supplementary Motor Area.
